# Ultrasonographic and elastographic evaluation of plantar fascia and Achilles tendon alterations in type 2 diabetes mellitus

**DOI:** 10.55730/1300-0144.6042

**Published:** 2025-06-23

**Authors:** Rıfat BOZKUŞ, İhsaniye SÜER DOĞAN, Berna TURHAN, Sümeyya DURAN KAYMAK, Mustafa Hulusi KURT, Rasime Pelin KAVAK

**Affiliations:** 1 Department of Internal Medicine, Ankara Etlik City Hospital, Ankara, Turkiye; 2 Department of Radiology, Ankara Etlik City Hospital, Ankara, Turkiye

**Keywords:** Type 2 diabetes mellitus, plantar fascia, Achilles tendon, ultrasonography, shear wave elastography

## Abstract

**Background/aim:**

The primary aim of this study was to compare the thickness and stiffness of the plantar fascia (PF) and Achilles tendon (AT) between healthy volunteers and patients with type 2 diabetes mellitus (T2DM). A secondary objective was to explore the correlations between the thickness and stiffness of the PF and AT and diabetes duration (in months) and hemoglobin A1c (HbA1c) levels in T2DM patients without peripheral neuropathy or foot ulcers, with peripheral neuropathy, or with foot ulcers.

**Material and methods:**

A total of 289 participants, including 117 healthy volunteers (Group A) and 172 T2DM patients (59.5%), matched for age, sex, and body mass index, were included. The T2DM cohort was stratified into three subgroups: Group B (without peripheral neuropathy or foot ulcers), Group C (with peripheral neuropathy), and Group D (with foot ulcers). Ultrasonography (USG) and shear wave elastography (SWE) were used to assess PF and AT properties, and correlations with diabetes duration and HbA1c levels were analyzed.

**Results:**

T2DM patients exhibited significantly greater PF and AT thickness, and lower stiffness compared to healthy controls (p < 0.001). Among subgroups, Group D had the greatest PF and AT thickness and the lowest stiffness, followed by Group C and then Group B (p < 0.001 for all comparisons). Positive correlations were observed between diabetes duration, HbA1c levels, and tendon thickness, while negative correlations were identified with stiffness (p < 0.001 for all).

**Conclusion:**

USG and SWE revealed significant alterations in PF and AT properties in T2DM patients compared to healthy individuals, with these changes correlating with diabetes severity and duration.

## 1. Introduction

Diabetes mellitus (DM) is a metabolic disorder characterized by chronic hyperglycemia, that affect a substantial proportion of the global population. Type 2 DM (T2DM) is the most prevalent form, accounting for approximately 90%–95% of all cases [1].

The plantar fascia (PF) and Achilles tendon (AT) are integral to foot biomechanics, functioning alongside the metatarsophalangeal joint to stabilize the medial longitudinal arch during propulsion and to prevent its collapse upon landing [2, 3]. The PF supports the medial longitudinal arch and facilitates propulsion, while the AT, known as the largest, thickest, and strongest tendon in the body, connects the calf muscles to the heel. Their mechanical properties are interdependent, modulating the passive range of motion of the ankle-foot complex and influencing stiffness and movement dynamics [4].

Hicks introduced the concept of the windlass mechanism, wherein dorsiflexion of the metatarsophalangeal joints elevates the arch, increasing tension and enhancing stability. The combined function of the PF and AT plays a critical role in maintaining arch integrity, regulating tension, and influencing foot mechanics [5]. This interplay also underlies the development of certain pathologies, such as plantar fasciitis [6].

DM exerts a profound impact on the biomechanical and anatomical properties of the PF and AT, contributing to the development of related pathologies. Persistent hyperglycemia leads to nonenzymatic glycosylation, resulting in the excessive accumulation of advanced glycation end-products (AGEs) within the PF and AT [7].

Ultrasonography (USG) serves as a reliable tool for evaluating tendon thickness, while shear wave elastography (SWE) provides precise measurements of tissue stiffness [8].

Due to the clinically heterogeneous nature of diabetic complications, this study stratified patients with T2DM into clinically meaningful subgroups: those without peripheral neuropathy or foot ulcers, those with peripheral neuropathy, and those with diabetic foot ulcers. This stratification allows for a more nuanced investigation of how varying degrees of disease severity and complication profiles influence tendon structure and mechanical properties. Patients with peripheral neuropathy may exhibit early microvascular and neural changes that subtly alter tendon integrity. At the same time, the presence of diabetic foot ulcers is often indicative of more advanced pathology involving chronic inflammation, tissue breakdown, and impaired healing. By analyzing these subgroups separately, the study aims to delineate the specific biomechanical and structural tendon alterations associated with each complication, which could otherwise be masked if diabetic patients were analyzed as a single, undifferentiated group.

The primary aim of this study was to compare the thickness and stiffness of the PF and AT between healthy volunteers and patients with T2DM. A secondary objective was to explore the correlations between the thickness and stiffness of the PF and AT and diabetes duration (in months) and hemoglobin A1c (HbA1c) levels in T2DM patients without peripheral neuropathy or foot ulcers, with peripheral neuropathy, or with foot ulcers.

This study hypothesized that patients with T2DM would exhibit significantly increased thickness but reduced stiffness in both the PF and AT compared to healthy individuals, and that these alterations would positively correlate with diabetes duration and HbA1c levels, particularly in those with neuropathy or foot ulcers.

## 2. Material and methods

### 2.1. Study population and patient evaluations

The study enrolled healthy volunteers aged 18 years and older (Group A) as well as patients with T2DM, matched with the control group for age, sex, and body mass index (BMI) (calculated as weight in kilograms divided by height in meters squared) (Table 1). The T2DM group was categorized into three subgroups: patients without peripheral neuropathy or foot ulcers (Group B), those with peripheral neuropathy (Group C), and those with foot ulcers (Group D).

Exclusion criteria included a history of foot trauma, previous foot or ankle surgeries, congenital ankle deformities, hallux valgus, other forms of diabetes, peripheral vascular diseases, rheumatoid disorders, active plantar fasciitis, nondiabetic foot ulcer, neurological diseases, surgical interventions involving the lower extremities, restricted ankle joint motion, musculoskeletal disorders, cognitive impairment, malignancy, cardiovascular conditions, or any other musculoskeletal or systemic issues that could influence balance or gait. The duration of diabetes and HbA1c levels of patients with T2DM were recorded.

Each participant was instructed to recline on the examination table in the prone position, ensuring that both feet hung freely over the edge of the table in a state of complete relaxation. Measurements of the thickness and stiffness (shear wave velocity) of the right and left PF and AT were performed by consensus among three experienced radiologists. These radiologists, blinded to the clinical status of the participants, utilized a LOGIQ E10 ultrasound system (GE Healthcare, Chicago, IL, USA) equipped with a linear probe.

**Figure 1:**
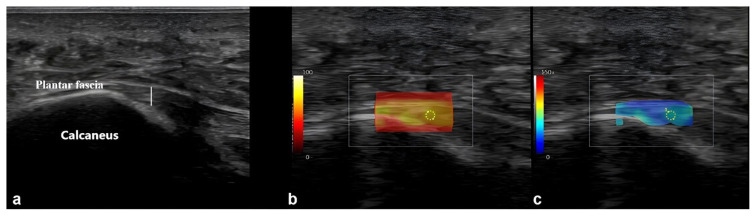
Ultrasonographic imaging sections showing measurements of the thickness (white line) (a), and stiffness (shear wave velocity) (b, c) of the plantar fascia.

The PF was assessed on the medial aspect of the foot, while the AT was evaluated at its midpoint, approximately 6 cm proximal to its insertion site. Measurements of anteroposterior diameters were obtained using transverse scans, with results documented in millimeters (mm) (Figures 1a, 1b, and 1c).

**Figure 2:**
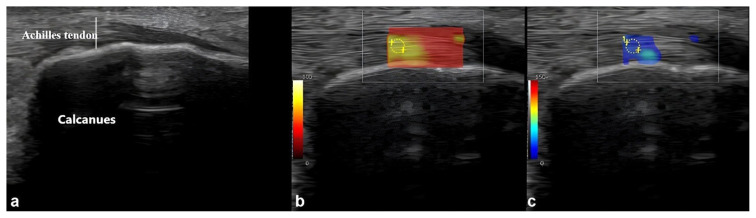
Ultrasonographic imaging sections showing measurements of the thickness (white line) (a), and stiffness (shear wave velocity) (b, c) of the Achilles tendon.

A circular region of interest (ROI) was placed on the PF and AT, and activated using the virtual touch quantification mode. The ROI was carefully adjusted to exclude adjacent structures and ensure precise measurements. At least four measurements were taken for each structure, and the mean stiffness was determined by averaging these values in kilopascals (kPa) (Figures 2a, 2b, and 2c).

### 2.2. Statistical analysis

Descriptive statistics for continuous variables were reported as mean ± standard deviation, median, minimum, and maximum values, whereas categorical variables were summarized as frequencies and percentages. The normality of continuous data was assessed using the Shapiro-Wilk test.

For nominal variables, group comparisons were conducted using the chi-square test. Continuous variables were compared between the DM and control groups using the Mann-Whitney U test. Comparisons of continuous variables among DM subgroups were performed using the Kruskal-Wallis analysis of variance. Post hoc evaluations to identify the source of significant differences were carried out with the Kruskal-Wallis multiple comparison test.

Relationships between continuous variables were analyzed using Spearman’s correlation coefficient. Statistical analyses were performed using IBM SPSS for Windows version 20.0 (IBM Corp., Armonk, NY, USA, with a p-value of < 0.05 considered indicative of statistical significance.

## 3. Results

The study included 289 participants, comprising 117 healthy individuals (Group A, 40.5%) and 172 patients with T2DM (59.5%).

Compared to Group A, patients with T2DM demonstrated significantly increased thickness of the right and left PF and AT (p < 0.001 for both). Furthermore, elastography values of the right and left PF and AT were significantly reduced in T2DM patients compared to Group A (p < 0.001 for both) (Table 2).

Within the T2DM group, 72 patients (41.9%) were classified as Group B, 60 patients (34.9%) as Group C, and 40 patients (23.2%) as Group D. The thickness of the right and left PF and AT was highest in Group D, followed by Group C, and lowest in Group B (p < 0.001 for all comparisons). Similarly, shear wave elastography imaging (SWEI) values of the right and left PF and AT were lowest in Group D, followed by Group C, and highest in Group B (p < 0.001 for all comparisons) (Table 3).

In Groups B, C, and D, a significant positive correlation was identified between the duration of diabetes (in months) and the thickness of the right and left PF and AT (p < 0.001 for both). In contrast, a significant negative correlation was observed between the duration of diabetes and the elastography values of the right and left PF and AT (p < 0.001 for both) (Table 4). Similarly, in Groups B, C, and D, HbA1c levels demonstrated a positive correlation with the thickness of the right and left PF and AT (p < 0.001 for both). Conversely, HbA1c levels were negatively correlated with the elastography values of the right and left PF and AT (p < 0.001 for both) (Table 5).

In the multivariate linear regression analysis, age, BMI, AT thickness, PF elasticity, and AT elasticity were identified as significant predictors of HbA1c levels in the T2DM group (R^2^ = 0.959, F = 214.968, p < 0.001). In Group C, age, BMI, and PF thickness were found to be the significant predictors of HbA1c levels (R^2^ = 0.974, F = 273.245, p < 0.001). In Group D, PF thickness, PF elasticity, and AT elasticity were found to be the significant predictors of HbA1c levels (R^2^ = 0.984, F = 279.280, p < 0.001) (Table 6).

In the multivariate linear regression analysis, BMI, PF thickness, and PF elasticity were identified as significant predictors of diabetes duration in the T2DM group (R^2^ = 0.903, F = 85.485, p < 0.001). In Group D, AT thickness was identified as a significant predictor of disease duration (R^2^ = 0.952, F = 90.140, p < 0.001) (Table 7).

## 4. Discussion

DM induces several pathological mechanisms that adversely affect tendon structure and function. The accumulation of AGEs in tendon tissues leads to cross-linking between collagen fibers, increasing stiffness while reducing elasticity. Furthermore, oxidative stress and inflammatory pathways exacerbate these changes, disrupting normal tendon homeostasis. Microvascular impairments, such as reduced blood flow and nutrient delivery, further compound these effects by limiting the tendons’ ability to repair and remodel over time. These combined mechanisms explain the structural and functional alterations observed in the PF and AT in patients with DM. The structural and functional changes in tendons due to DM have significant clinical consequences. Diabetic patients are at a higher risk of developing tendinopathies, leading to pain, restricted mobility, and an increased likelihood of tendon ruptures. These musculoskeletal complications can further reduce physical activity levels, worsening glycemic control and overall health outcomes.

Diabetic peripheral neuropathy and foot ulcers are significant complications of DM, often leading to severe outcomes such as infections and amputations. Foot ulcers arise from the combination of neuropathy, peripheral vascular disease, and biomechanical abnormalities. Effective management and prevention strategies are crucial to mitigate these risks. This highlights the critical need for early identification and management of tendon alterations in diabetic patients to prevent progression and improve their quality of life.

Different diabetic treatment regimens, such as insulin therapy, oral hypoglycemic agents, and lifestyle modifications, may impact tendon health in unique ways. Emerging evidence suggests that certain treatments, like insulin therapy, might influence tendon properties, although the precise mechanisms are not fully understood. Understanding the effects of these treatments on tendon tissues is essential for designing holistic management plans that address both metabolic control and musculoskeletal health in diabetic patients.

In this study, patients with T2DM exhibited increased thickness and decreased stiffness of the right and left PF and AT compared to the control group.

Consistent with the study findings, previous research has reported greater PF and AT thickness in diabetic patients than in nondiabetic controls [3, 8, 9, 10]. Huang et al. observed reduced Young’s modulus values in diabetic patients, indicative of diminished stiffness across various tendon regions [11]. Similarly, Saroha et al. identified thickening and softening of the PF and AT in T2DM patients compared to healthy controls, as evaluated using USG and SWE [10].

Nonenzymatic glycosylation, a defining feature of persistent hyperglycemia in diabetes, leads to the excessive deposition of AGEs [7]. In diabetic individuals, collagen glycation increases tissue stiffness, potentially disrupting gait dynamics and elevating plantar pressure. This process involves the formation of AGEs that create cross-links between collagen molecules, enhancing the structural rigidity of collagen fibrils. These cross-links increase stiffness while reducing the flexibility and remodeling capacity of the fibrils [12]. The accumulation of AGEs in the PF and AT contributes to tissue thickening and increased vascularization [13]. Diabetic peripheral neuropathy intensifies pathological changes in the AT and PF, further reducing tendon elongation capacity and increasing stiffness, which can significantly disrupt gait mechanics [14]. The thickening and altered mechanical properties of the PF and AT compromise the windlass mechanism, a key function in foot propulsion during walking. This dysfunction elevates plantar pressures and alters foot biomechanics, thereby increasing the risk of developing foot ulcers [15]. Furthermore, diabetic vasculopathy contributes to microcirculatory dysfunction, characterized by endothelial impairment and reduced secretion of vasodilators, which results in ischemia and delays in foot ulcer healing [16]. Evranos et al., the first to assess the AT in diabetic patients using strain elastography, reported a significant decrease in AT stiffness in patients with foot ulcers compared to healthy controls.They demonstrated that, while no significant differences were observed in the proximal one-third of the tendon among the groups, the medial and distal thirds showed notable softening in diabetic patients with foot ulcers [17]. In this study, the elastography values of the right and left PF and AT were noticeably lower in the foot ulcer group (Group D) compared to the peripheral neuropathy group (Group C) and the T2DM group without peripheral neuropathy or foot ulcers (Group B). Multiple studies have reported significant tendon alterations in DM patients compared to healthy controls. One study demonstrated an increase in tendon thickness among DM patients and identified correlations between these changes and diabetes duration as well as HbA1c levels, indicating that chronic hyperglycemia contributes to the thickening and stiffness of the PF and AT [18]. Afolabi et al., in a study involving 160 participants including 80 patients with T2DM and 80 age and sex matched nondiabetic controls reported a weak but significant positive correlation between diabetes duration and increased thickness of the PF and AT, suggesting that although the observed association was not strong, prolonged hyperglycemia may contribute to a tendency toward increased tendon thickness [7]. Teoh et al., in their study involving 70 diabetic patients and lacking a control group, observed that increased plantar tissue stiffness was associated with longer diabetes duration, higher BMI, and poor glycemic control (HbA1c > 10%); this indicates that the study was specifically designed to investigate the relationship between these clinical parameters and plantar tissue stiffness within a diabetic population [19]. In contrast, Saroha et al. found no statistically significant correlation between the thickness and stiffness of the AT and PF and factors such as patient age, diabetes duration, or HbA1c levels, although some trends were observed [10]. A relatively small sample size (55 patients with T2DM and 55 healthy volunteers) may not provide sufficient statistical power to detect subtle associations between AT and PF characteristics and clinical variables. Notably, these studies did not differentiate between patients with and without peripheral neuropathy or include those with foot ulcers. By not distinguishing between these groups, the study was unable to determine whether neuropathy contributes to or modifies the observed changes in PF and AT. In this study, a positive correlation was observed between the thickness of the right and left PF and AT and HbA1c levels, the presence of foot ulcers (Group D), and peripheral neuropathy (Group C). Conversely, a negative correlation was identified between the stiffness of the right and left PF and AT and these variables. Compared to previous studies, the present study demonstrates a methodological advantage by including a well-defined control group and stratifying diabetic patients into clinically meaningful subgroups (with and without peripheral neuropathy and foot ulcers). Furthermore, the simultaneous assessment of both tendon thickness and stiffness regarding glycemic control, neuropathy, and ulceration provides a more comprehensive understanding of tendon pathology in diabetes. The observed positive correlation between tendon thickness and clinical severity markers, alongside a negative correlation with stiffness, highlights the structural-functional dissociation that may not have been fully captured in earlier studies.

The duration of T2DM and elevated HbA1c levels, markers of poor glycemic control, are significant predictors of tendon changes associated with diabetes. As a chronic condition, DM exerts cumulative effects over time, making disease duration a critical factor in evaluating its impact. Prolonged hyperglycemia likely contributes to gradual alterations in tissue properties, underscoring the importance of long-term exposure in understanding these changes.

The multivariable regression analyses conducted in the study revealed that HbA1c levels were significantly associated with various structural and functional tendon parameters across different clinical subgroups of T2DM patients.

Among patients with T2DM who had not developed neuropathy or ulcers, the variables that significantly influenced HbA1c levels included age, BMI, AT thickness, and the elastography values of both the PF and AT. This finding suggests that the level of glycemic control is closely associated not only with metabolic factors but also with structural alterations in tendon tissues.

In patients with peripheral neuropathy, the factors most strongly associated with HbA1c levels were identified as age, BMI, and PF thickness. This suggests that in individuals with established nerve damage, structural changes in the tendon may better reflect metabolic control. In patients with foot ulcers, HbA1c levels were found to be negatively associated with both the thickness of the PF and the elasticity parameters of the PF and AT. These findings support the notion that advanced-stage complications severely alter both the stiffness and structure of tendons, making them directly related to glycemic dysregulation. Similarly, regression analyses performed on disease duration revealed that tendon structures had significant explanatory power across different clinical subgroups. In patients with T2DM who had not developed neuropathy or ulcers, disease duration was significantly associated with BMI, PF thickness, and its elasticity values. This finding indicates that increased body weight and structural or biomechanical changes in the PF tend to parallel the progression of diabetes over time. On the other hand, in patients with foot ulcers, the most powerful predictor of disease duration was identified as AT thickness. This result suggests that as the duration of diabetes increases, a marked thickening of the AT occurs, which may be linked to the development of severe complications such as diabetic foot.

USG and SWE are imaging modalities whose accuracy depends heavily on the operator’s expertise, knowledge, and technical skill. A key strength of this study is the standardized protocol employed, whereby all USG and SWE assessments were conducted by three experienced radiologists with specialized training in musculoskeletal ultrasonography and elastography. These radiologists, blinded to the participants’ clinical information, performed the evaluations collaboratively, ensuring consistency and enhancing the validity of the findings.

One limitation of this study is the variability in diabetic treatment regimens among the enrolled patients, which may have influenced the results. Due to the retrospective design and the absence of standardized diabetes treatment protocols, patients were not categorized based on their treatment regimens. Although experienced radiologists performed the evaluations, the lack of an intraclass correlation coefficient (ICC) assessment represents a limitation in terms of interobserver and intraobserver reliability. Another limitation of this study is the lack of adjustment for potential confounding factors, such as diabetes treatment regimens and disease duration. Both glycemic control strategies and disease chronicity can affect tendon microstructure, stiffness, and elasticity through mechanisms such as AGEs, collagen cross-linking, and chronic inflammation. Therefore, it is recommended that future studies incorporate these variables either through multivariate adjustment or detailed subgroup analyses.

## 5. Conclusion

This study demonstrates significant alterations in the PF and AT of patients with T2DM, characterized by increased thickness and reduced stiffness. These changes were positively correlated with HbA1c levels and diabetes duration, with greater deviations observed in patients with peripheral neuropathy and foot ulcers. Such alterations, likely driven by chronic hyperglycemia and the accumulation of AGEs, impair tendon elasticity and foot biomechanics, contributing to complications such as increased plantar pressures and diabetic foot ulcers. Microvascular impairments in T2DM further exacerbate these changes by reducing tissue perfusion and delaying wound healing.

Clinically, these findings highlight the importance of early screening and monitoring of tendon health in T2DM patients using USG and SWE. These techniques offer valuable insights into musculoskeletal changes and can guide interventions to improve glycemic control, reduce biomechanical stress, and mitigate the risk of complications. Future research should focus on the long-term effects of different diabetes treatments on tendon properties and the potential of USG and SWE as predictive tools for diabetic complications, enabling a more holistic approach to patient management.

## Figures and Tables

**Table 1:** Comparison of age, body mass index (BMI), and sex between Group A and patients with type 2 diabetes mellitus (T2DM).

	Group A (n = 117)	Type 2 DM patients (n = 172)	p-value
**Age (year) (Mean± SD)**	58.72 ± 5.03	59.79 ± 3.95	0.129 b
**Median (min-max)**	60 (49–66)	60 (49–66)	
**Sex, n (%)**			
Female	58 (49.6)	100 (58.1)	0.151 c
Male	59 (50.4)	72 (41.9)	
**Body mass index (kg/m** **^2^** **) (Mean± SD)** **Median (min–max)**	27.11 ± 3.52 26 (23–34.5)	27.57 ± 4.29 27.5 (20–36.5)	0.327 b

b Mann Whitney U test;

c chi-Square test;

Type 2 DM: type 2 diabetes mellitus; SD: standard deviation; kg/m^2^: kilogram/square meter; min: minimum; max: maximum.

**Table 2:** Comparison of plantar fascia (PF) and Achilles tendon (AT) thickness, and stiffness between Group A and patients with type 2 diabetes mellitus (T2DM).

	Group A (n = 117) Mean ± SD	Type 2 DM patients (n = 172) Mean ± SD	p-value
**Right thickness of PF (mm)**	1.78 ± 0.19	3.07 ± 0.55	**<0.001** b
**Left thickness of PF (mm)**	1.77 ± 0.15	3.07 ± 0.56	**<0.001** b
**Right thickness of AT (mm)**	4.37 ± 0.30	5.83 ± 0.33	**<0.001** b
**Left thickness of AT (mm)**	4.34 ± 0.32	5.84 ± 0.31	**<0.001** b
**Right PF stiffness (kPa)**	6.28 ± 0.01	3.23 ± 0.87	**<0.001** b
**Left PF stiffness (kPa)**	6.28 ± 0.01	3.22 ± 0.87	**<0.001** b
**Right AT stiffness (kPa)**	7.26 ± 0.03	4.13 ± 0.95	**<0.001** b
**Left AT stiffness (kPa)**	7.26 ± 0.03	4.12 ± 0.95	**<0.001** b

b Mann Whitney U test;

PF: plantar fascia; AT: Achilles tendon; mm: millimeter; kPa: kilopascal; Type 2 DM: type 2 diabetes mellitus; SD: standard deviation.

**Table 3:** Comparison of plantar fascia (PF) and Achilles tendon (AT) thickness, and stiffness among Groups B, C, and D.

	Group B (n = 72)	Group C (n = 60)	Group D (n = 40)	p-value
	Mean± SD	Mean± SD	Mean± SD	
**Right thickness of PF (mm)**	2.63 ± 0.24	3.07 ± 0.15	3.87 ± 0.43	**<0.001** ^d^
**Post hoc**	a–b p < 0.001 a–c p < 0.001 b–c p < 0.001			
**Left thickness of PF (mm)**	2.62 ± 0.24	3.07 ± 0.15	3.87 ± 0.44	**<0.001** ^d^
**Post hoc**	a–b p < 0.001 a–c p < 0.001 b–c p < 0.001			
**Right thickness of AT (mm)**	5.87 ± 0.23	5.84 ± 0.08	6.28 ± 0.21	**<0.001** ^d^
**Post hoc**	a–b p < 0.001 a–c p < 0.001 b–c p < 0.001			
**Left thickness of AT (mm)**	5.59 ± 0.21	5.84 ± 0.07	6.27 ± 0.21	**<0.001** ^d^
**Post hoc**	a–b p < 0.001 a–c p < 0.001 b–c p < 0.001			
**Right PF stiffness (kPa)**	4.09 ± 0.67	2.79 ± 0.05	2.32 ± 0.20	**<0.001** ^d^
**Post hoc**	a–b p < 0.001 a–c p < 0.001 b–c p < 0.001			
**Left PF stiffness (kPa)**	4.09 ± 0.67	2.79 ± 0.05	2.32 ± 0.21	**<0.001** ^d^
**Post hoc**	a–b p < 0.001 a–c p < 0.001 b–c p < 0.001			
**Right AT stiffness (kPa)**	4.88 ± 0.67	4.10 ± 0.21	2.81 ± 0.45	**<0.001** ^d^
**Post hoc**	a–b p < 0.001 a–c p < 0.001 b–c p < 0.001			
**Left AT stiffness (kPa)**	4.88 ± 0.68	4.07 ± 0.22	2.81 ± 0.45	**<0.001** ^d^
**Post hoc**	a–b p < 0.001 a–c p < 0.001 b–c p < 0.001			

* Kruskal-Wallis test;

PF: plantar fascia; AT: Achilles tendon; mm: millimeter; kPa: kilopascal; SD: standard deviation.

**Table 4:** Correlations between diabetes duration (in months) and the thickness and stiffness of the right and left plantar fascia (PF) and Achilles tendon (AT) in Groups B, C, and D.

	Group B (n = 72)		Group C (n = 60)		Group D (n = 40)	
	Duration (months)		Duration (months)		Duration (months)	
	r*	p				
**Right thickness of PF (mm)**	0.961	<0.001	0.993	<0.001	0.997	<0.001
**Left thickness of PF (mm)**	0.952	<0.001	0.983	<0.001	0.997	<0.001
**Right thickness of AT (mm)**	0.957	<0.001	0.993	<0.001	0.996	<0.001
**Left thickness of AT (mm)**	0.955	<0.001	0.992	<0.001	0.993	<0.001
**Right PF stiffness (kPa)**	−0.954	<0.001	−0.985	<0.001	−0.995	<0.001
**Left PF stiffness (kPa)**	−0.958	<0.001	−0.985	<0.001	−0.995	<0.001
**Right AT stiffness (kPa)**	−0.959	<0.001	−0.989	<0.001	−0.938	<0.001
**Left AT stiffness (kPa)**	−0.956	<0.001	−0.986	<0.001	−0.928	<0.001

* r: Spearman’s correlation coefficient;

PF: plantar fascia; AT: Achilles tendon; mm: millimeter;kPa: kilopascal.

**Table 5:** Correlations between hemoglobin A1c (HbA1c) levels and the thickness and stiffness of the right and left plantar fascia (PF) and Achilles tendon (AT) in Groups B, C, and D.

	Group B		Group C		Group D	
	HbA1c		HbA1c		HbA1c	
	r*	p	r*	p	r*	p
**Right thickness of PF (mm)**	0.993	<0.001	0.985	<0.001	0.996	<0.001
**Left thickness of PF (mm)**	0.984	<0.001	0.970	<0.001	0.995	<0.001
**Right thickness of AT (mm)**	0.979	<0.001	0.987	<0.001	0.996	<0.001
**Left thickness of AT (mm)**	0.954	<0.001	0.986	<0.001	0.993	<0.001
**Right PF stiffness (kPa)**	−0.990	<0.001	−0.984	<0.001	−0.994	<0.001
**Left PF stiffness (kPa)**	−0.992	<0.001	−0.985	<0.001	−0.993	<0.001
**Right AT stiffness (kPa)**	−0.990	<0.001	−0.987	<0.001	−0.944	<0.001
**Right AT stiffness (kPa)**	−0.990	<0.001	−0.985	<0.001	−0.930	<0.001

* r: Spearman’s correlation coefficient;

HbA1c: hemoglobin A1c; PF: plantar fascia; AT: Achilles tendon; mm: millimeter; kPa: kilopascal.

**Table 6:** Results of the multivariate linear regression analysis identifying independent predictors of hemoglobin A1c (HbA1c) levels in Groups B, C, and D.

	B	Std. Error	95% CI		p-value
**Group B**					
Age (year)	0.041	0.020	0.001	0.081	**0.043**
Sex	0.020	0.020	−0.021	0.060	0.343
Body mass index (kg/m^2^)	0.056	0.013	0.031	0.081	**<0.001**
Thickness of PF (mm)	−0.061	0.248	−0.555	0.434	0.807
Thickness of AT (mm)	−0.343	0.138	−0.618	−0.068	**0.015**
PF stiffness (kPa)	−0.278	0.035	−0.348	−0.208	**<0.001**
AT stiffness (kPa)	0.156	0.067	0.023	0.290	**0.023**
**Group C**					
Age (year)	0.024	0.009	0.006	0.042	**0.010**
Sex (female)	0.011	0.009	−0.008	0.030	0.237
Body mass index (kg/m^2^)	−0.018	0.008	−0.035	−0.001	**0.035**
Thickness of PF (mm)	0.680	0.152	0.379	0.985	**<0.001**
Thickness of AT (mm)	0.719	0.532	−0.349	1.787	0.183
PF stiffness (kPa)	0.156	0.457	−0.761	1.073	0.735
AT stiffness (kPa)	0.028	0.139	−0.251	0.307	0.839
**Group D**					
Age (year)	−0.087	0.054	−0.197	0.022	0.114
Sex (female)	0.057	0.074	−0.093	0.207	0.444
Body mass index (kg/m^2^)	0.062	0.065	−0.071	0.195	0.352
Thickness of PF (mm)	2.016	0.365	1.272	2.759	**<0.001**
Thickness of AT (mm)	−1.676	0.784	−3.272	−0.080	**0.040**
PF stiffness (kPa)	−2.434	0.964	−4.398	−0.470	**0.017**
AT stiffness (kPa)	−0.194	0.222	−0.649	0.256	0.383

CI: confidence interval; PF: plantar fascia; AT: Achilles tendon; mm: millimeter; kPa: kilopascal; kg/m^2^: kilogram/square meter.

**Table 7:** Results of the multivariate linear regression analysis identifying independent predictors of diabetes duration in Groups B, C, and D.

	B	Std. Error	95% CI		p-value
**Group B**					
Age (year)	1.081	1.140	−1.197	3.358	0.347
Sex (female)	0.495	1.163	−1.829	2.819	0.672
Body mass index (kg/m^2^)	2.030	0.718	0.595	3.464	**0.006**
Thickness of PF (mm)	−38.343	14.105	−66.520	−10.166	**0.008**
Thickness of AT (mm)	12.896	7.848	−2.782	28.573	0.105
PF stiffness (kPa)	−7.865	1.991	−11.848	−3.888	**<0.001**
AT stiffness (kPa)	−5.505	3.813	−13.123	2.112	0.154
**Group C**					
Age (year)	1.272	0.818	−0.358	2.902	0.123
Sex (female)	0.304	0.837	−1.375	−1.982	0.718
Body mass index (kg/m^2^)	0.905	0.750	−0.599	2.409	0.233
Thickness of PF (mm)	4.463	13.620	−22.868	31.794	0.744
Thickness of AT (mm)	−64.319	47.714	−160.064	31.426	0.183
PF stiffness (kPa)	−88.423	40.967	−170.629	−6.217	**0.036**
AT stiffness (kPa)	−46.279	12.460	−71.282	−21.277	**<0.001**
**Group D**					
Age (year)	−0.062	0.929	−1.953	1.830	0.947
Sex	0.491	1.275	−2.107	3.088	0.703
Body mass index (kg/m^2^)	2.108	1.129	−0.192	4.408	0.071
Thickness of PF (mm)	−5.079	6.307	−17.927	7.768	0.427
Thickness of AT (mm)	−37.157	13.548	−64.753	−9.561	**0.010**
PF stiffness (kPa)	−55.756	16.673	−89.718	−21.794	**0.002**
AT stiffness (kPa)	−0.875	3.842	−8.700	6.950	0.821

CI: confidence interval; PF: plantar fascia; AT: Achilles tendon; mm: millimeter; kPa: kilopascal; kg/m^2^: kilogram/square meter.
